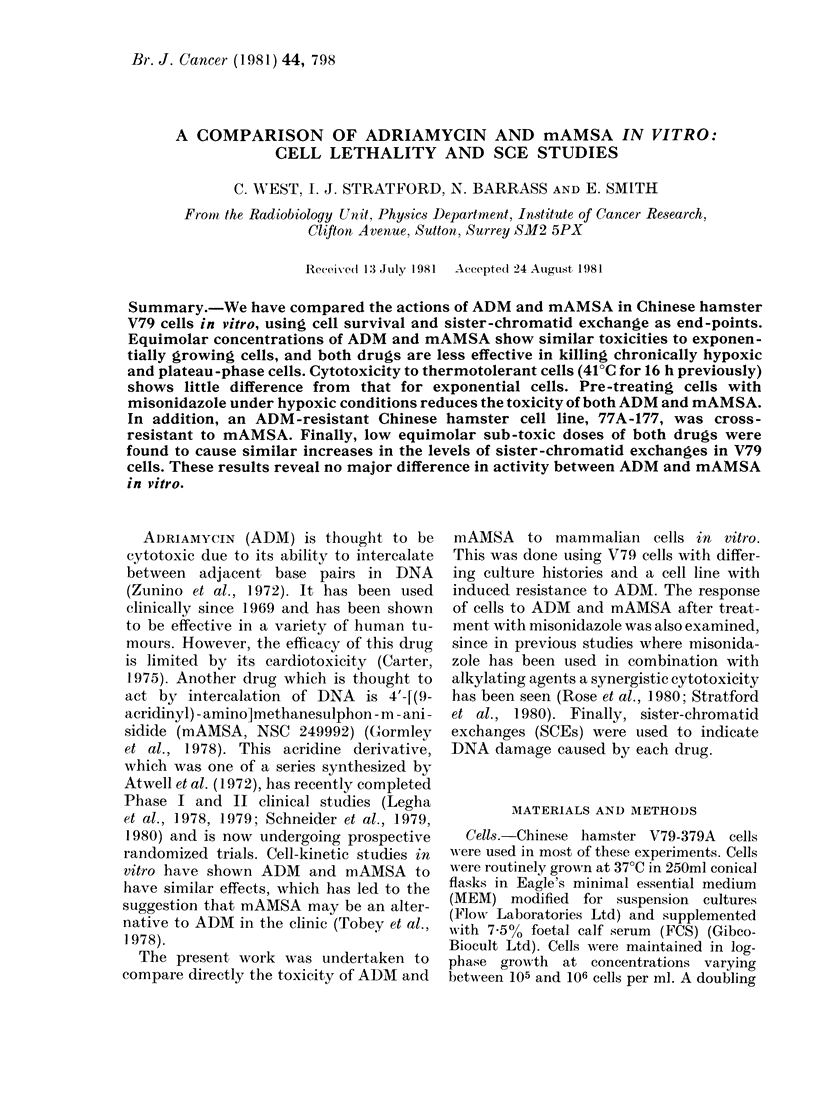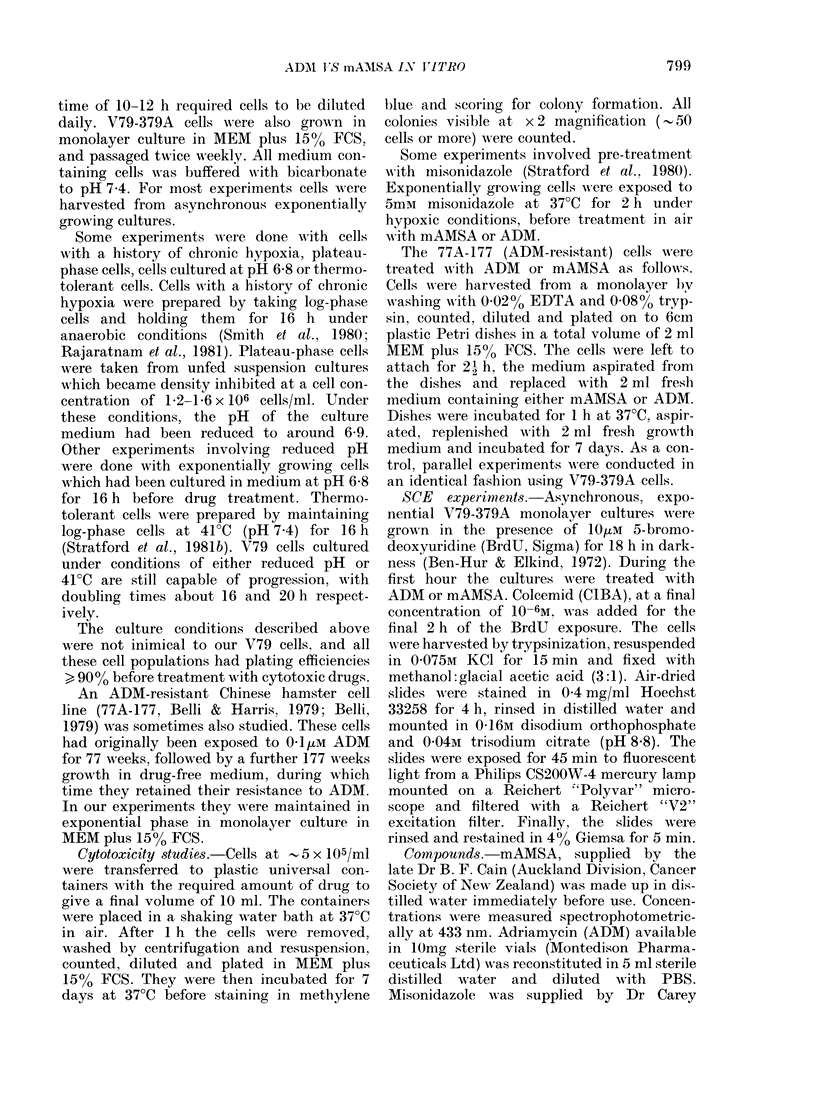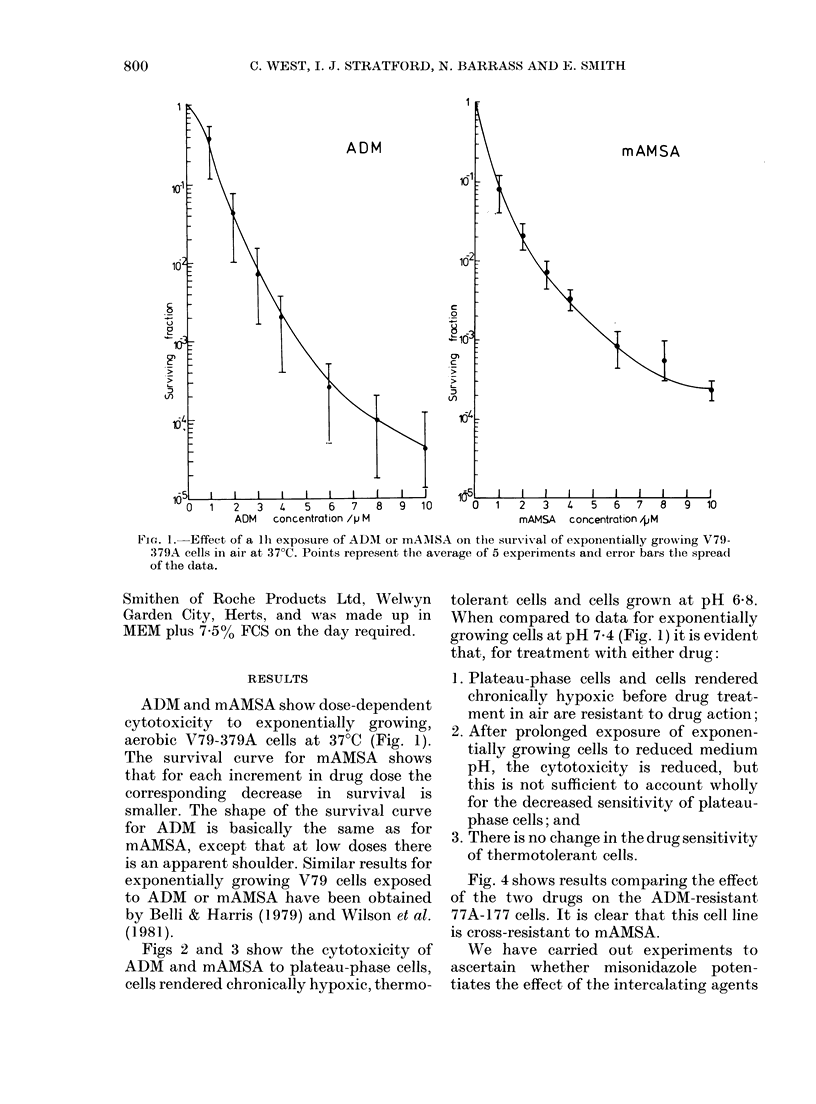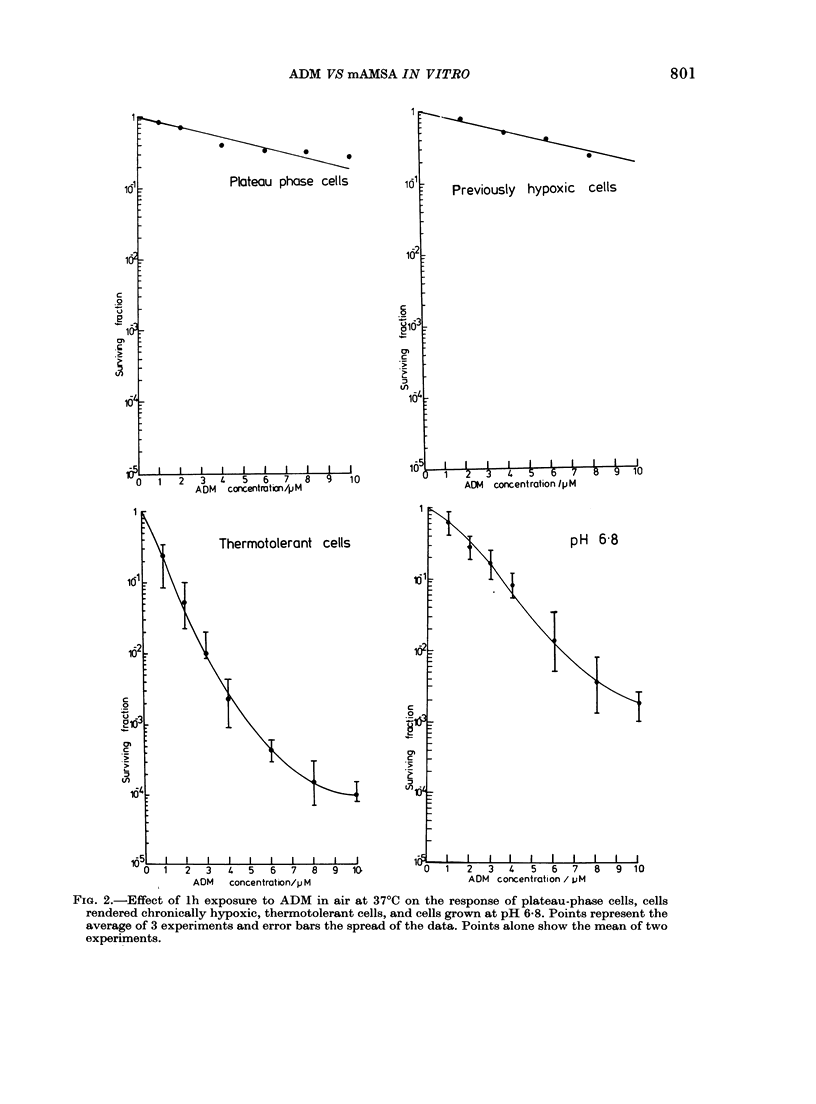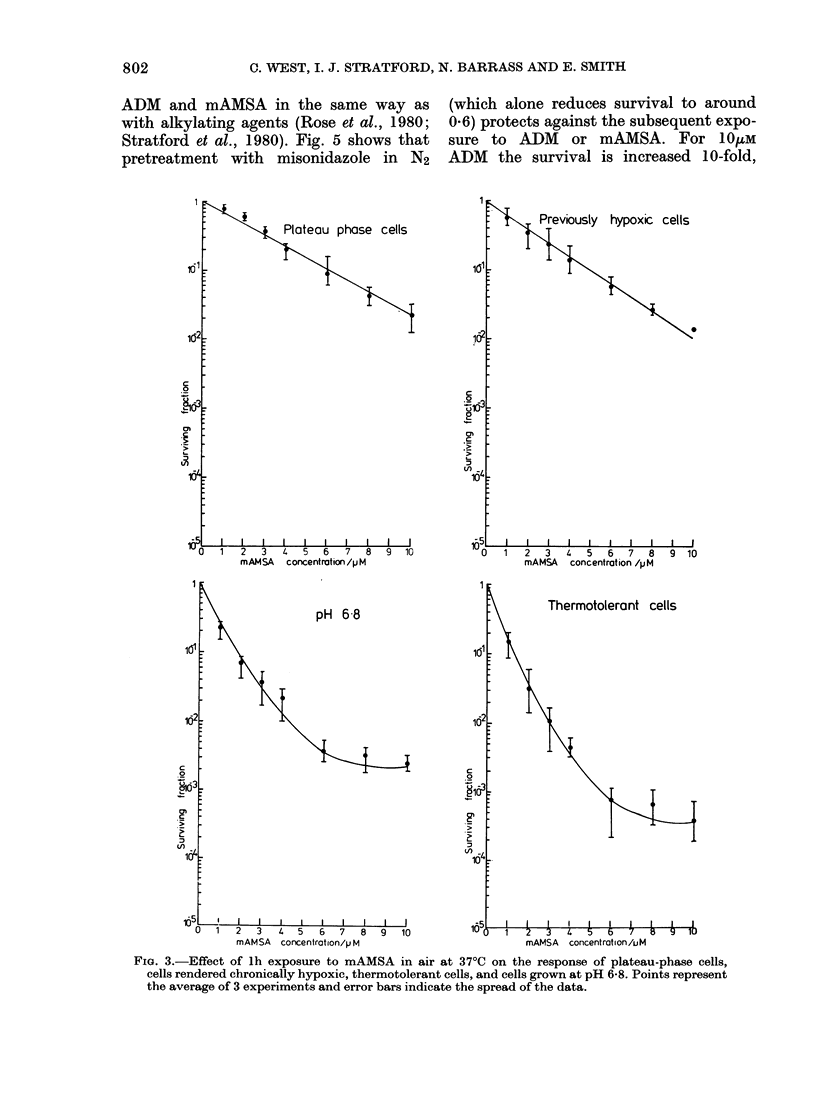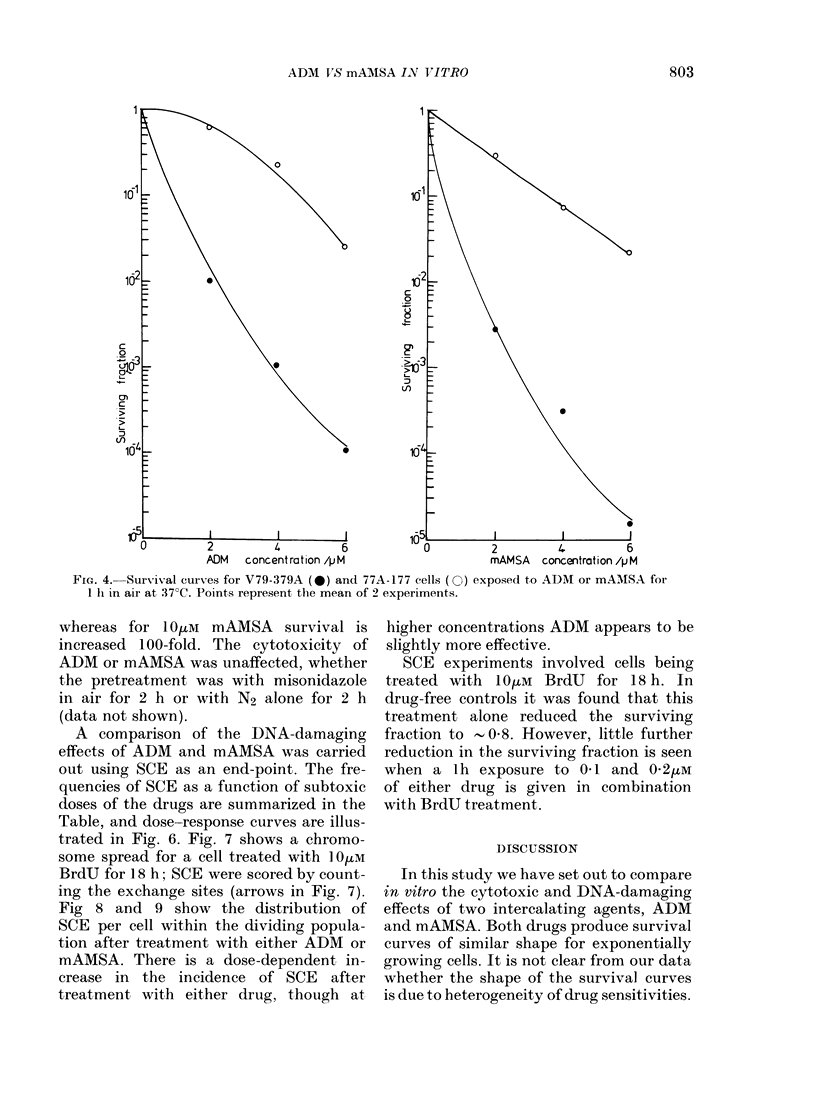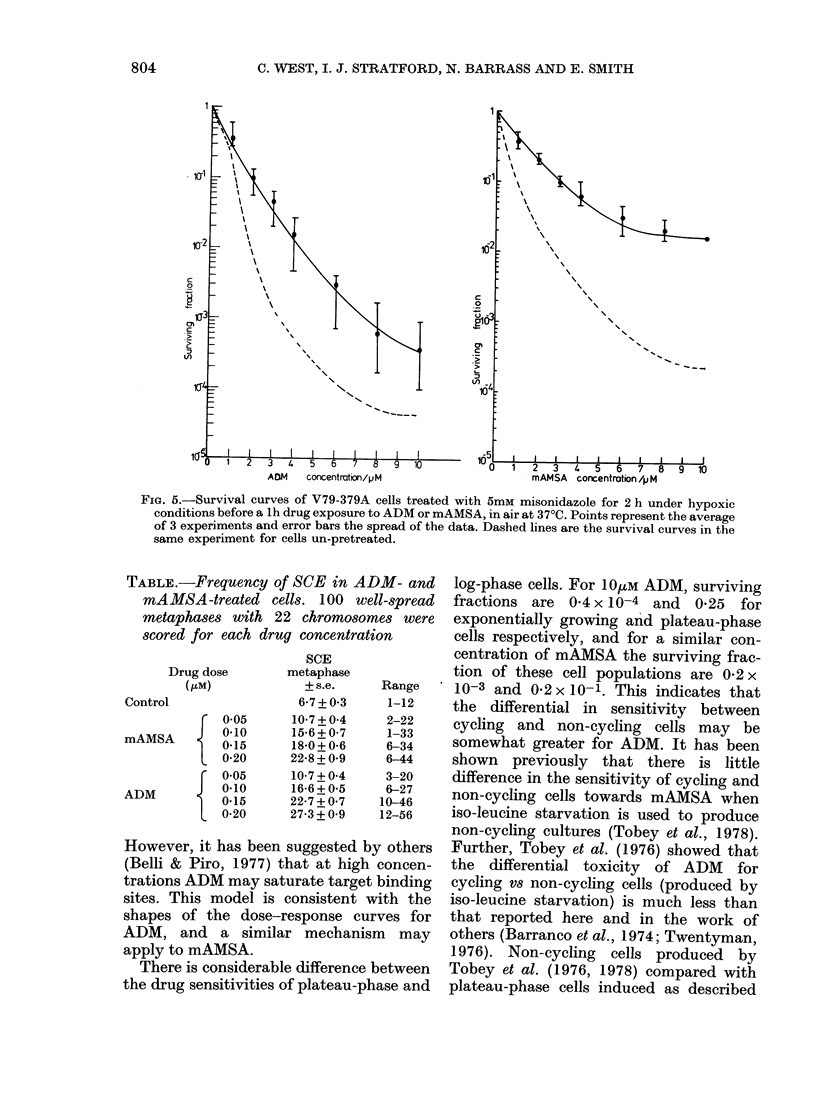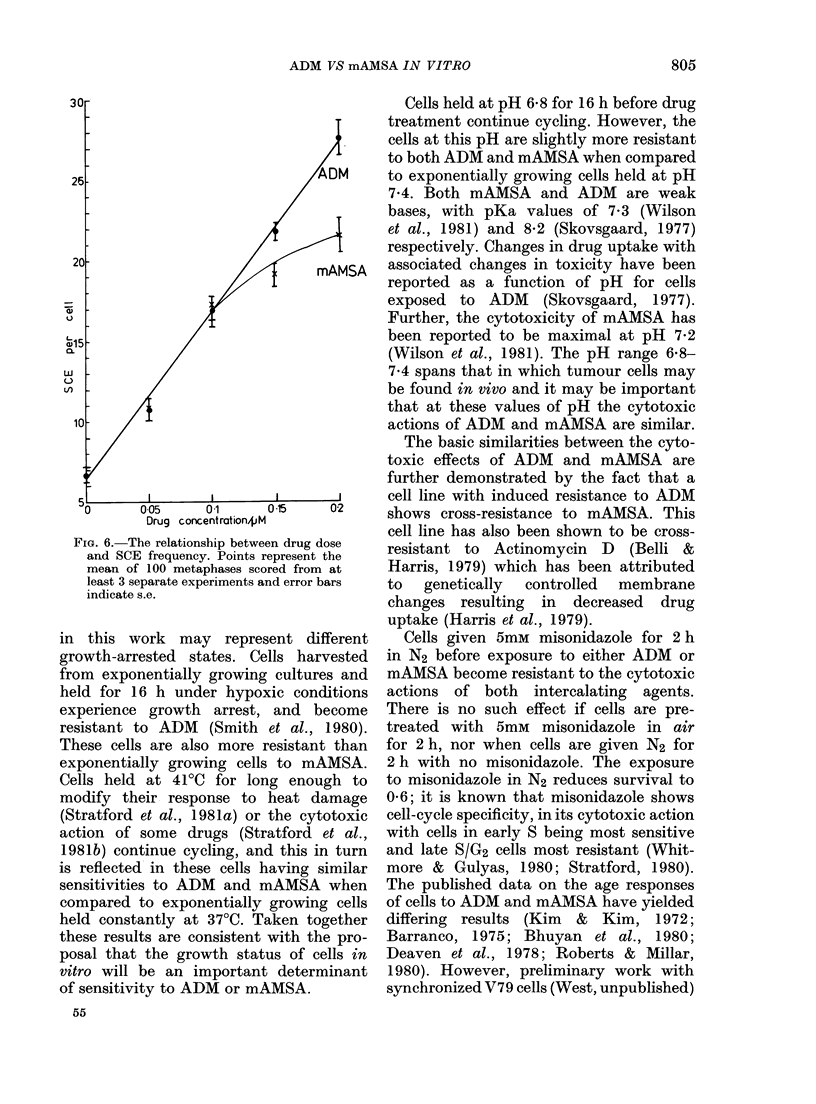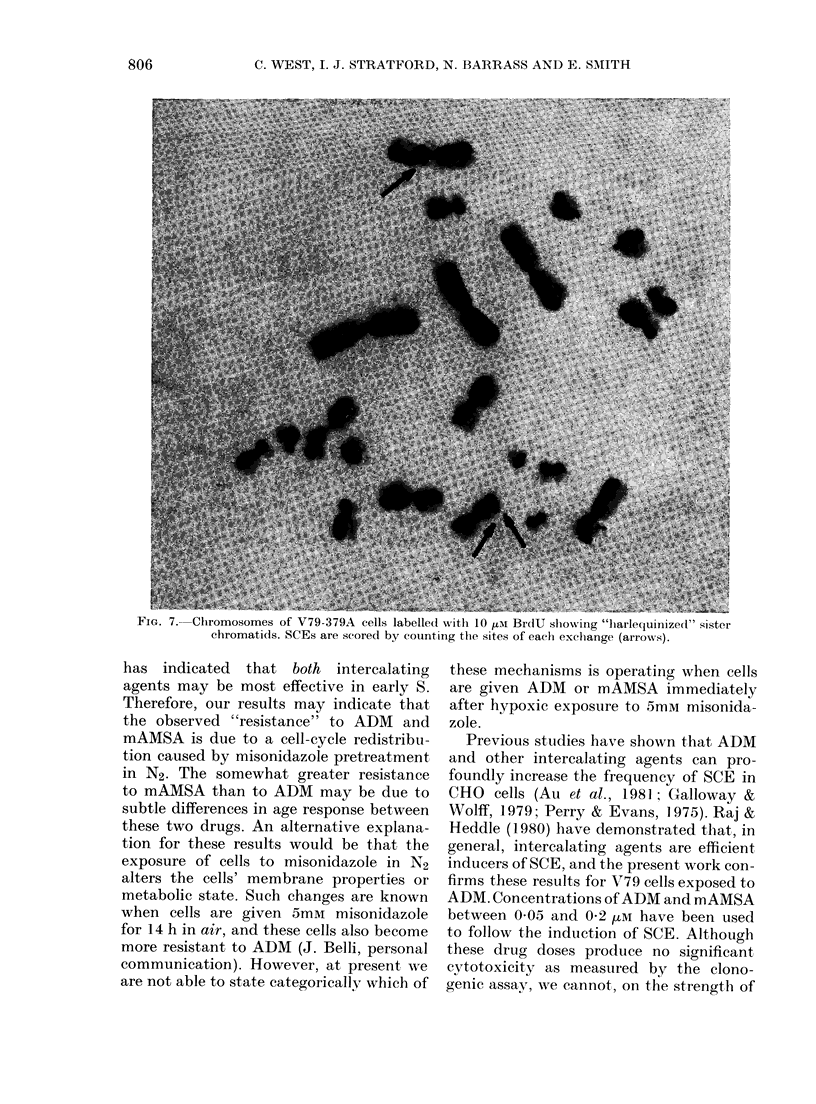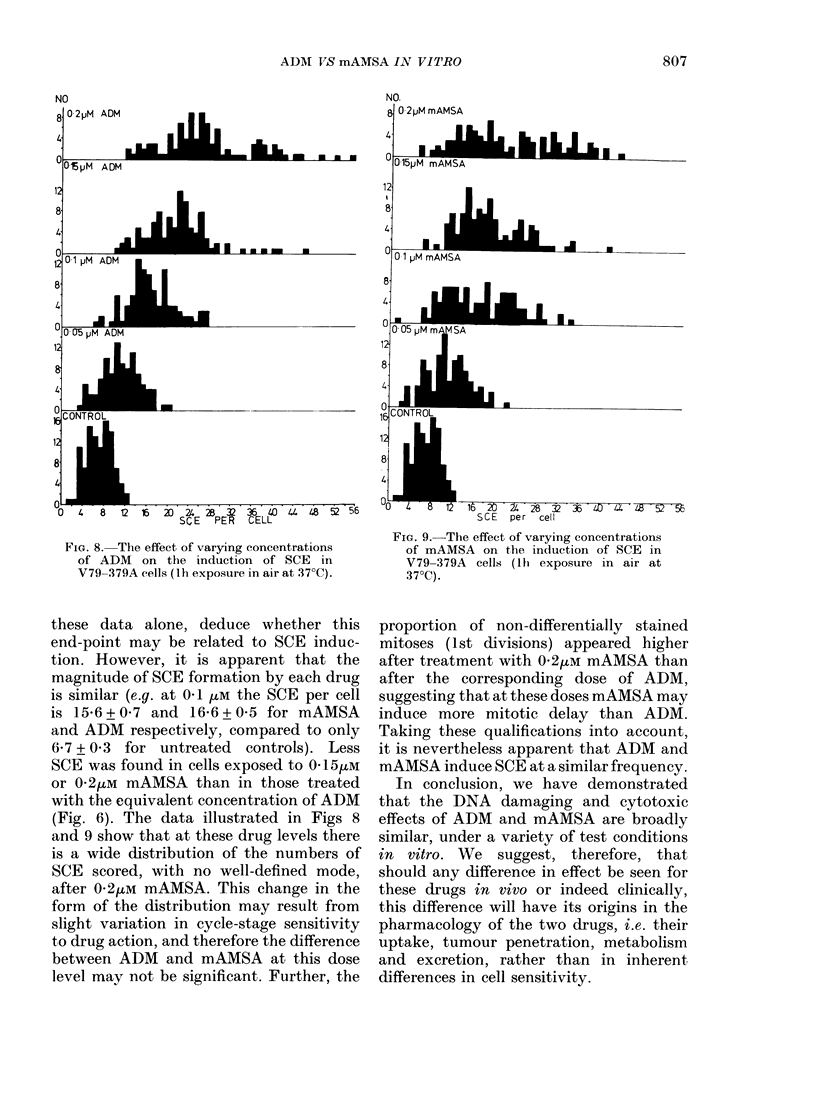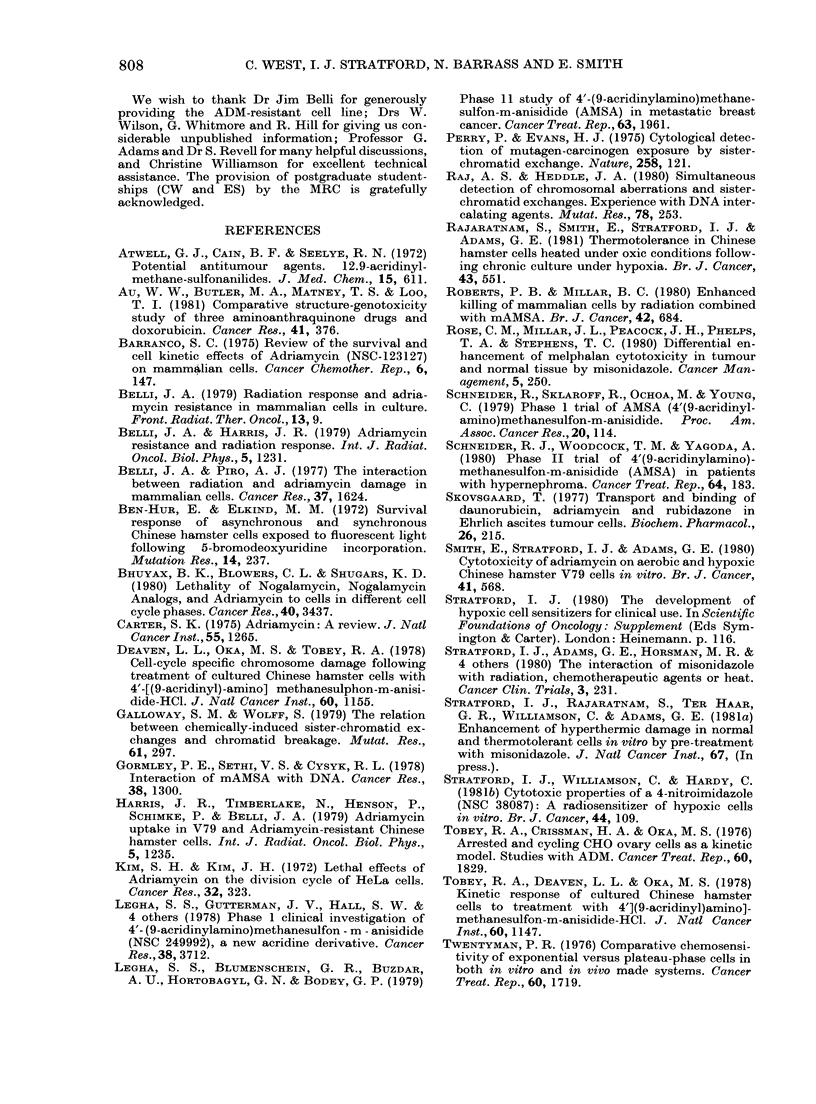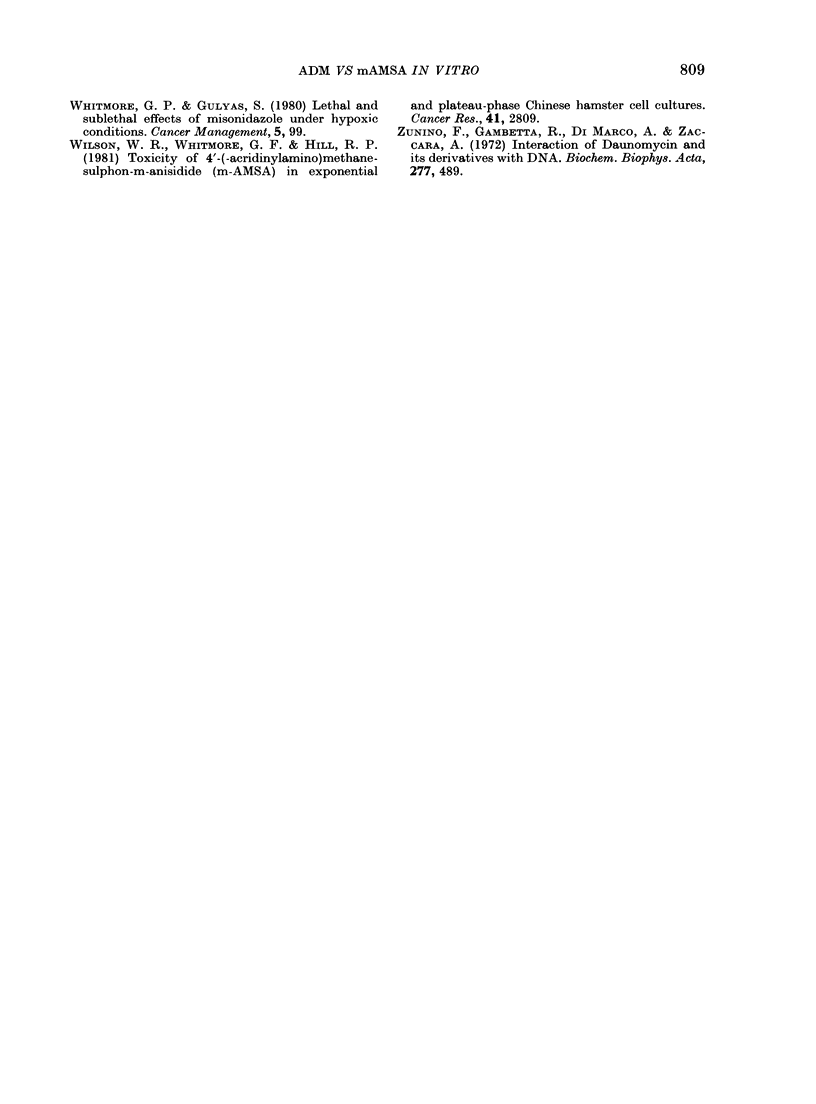# A comparison of adriamycin and mAMSA in vitro: cell lethality and SCE studies.

**DOI:** 10.1038/bjc.1981.278

**Published:** 1981-12

**Authors:** C. West, I. J. Stratford, N. Barrass, E. Smith

## Abstract

**Images:**


*Br. J. Cancer* (1981) **44**, 798

### A COMPARISON OF ADRIAMYCIN AND mAMSA *IN VITRO*: CELL LETHALITY AND SCE STUDIES

C. WEST, I. J. STRATFORD, N. BARRASS AND E. SMITH

*From the Radiobiology Unit, Physics Department, Institute of Cancer Research, Clifton Avenue, Sutton, Surrey SM2 5PX*

Received 13 July 1981 Accepted 24 August 1981

**Summary.—We have compared the actions of ADM and mAMSA in Chinese hamster V79 cells *in vitro*, using cell survival and sister-chromatid exchange as end-points. Equimolar concentrations of ADM and mAMSA show similar toxicities to exponentially growing cells, and both drugs are less effective in killing chronically hypoxic and plateau-phase cells. Cytotoxicity to thermotolerant cells (41°C for 16 h previously) shows little difference from that for exponential cells. Pre-treating cells with misonidazole under hypoxic conditions reduces the toxicity of both ADM and mAMSA. In addition, an ADM-resistant Chinese hamster cell line, 77A-177, was cross-resistant to mAMSA. Finally, low equimolar sub-toxic doses of both drugs were found to cause similar increases in the levels of sister-chromatid exchanges in V79 cells. These results reveal no major difference in activity between ADM and mAMSA *in vitro*.**

ADRIAMYCIN (ADM) is thought to be cytotoxic due to its ability to intercalate between adjacent base pairs in DNA (Zunino *et al.*, 1972). It has been used clinically since 1969 and has been shown to be effective in a variety of human tumours. However, the efficacy of this drug is limited by its cardiotoxicity (Carter, 1975). Another drug which is thought to act by intercalation of DNA is 4′-[(9-acridinyl)-amino]methanesulphon-m-anisidide (mAMSA, NSC 249992) (Gormley *et al.*, 1978). This acridine derivative, which was one of a series synthesized by Atwell *et al.* (1972), has recently completed Phase I and II clinical studies (Legha *et al.*, 1978, 1979; Schneider *et al.*, 1979, 1980) and is now undergoing prospective randomized trials. Cell-kinetic studies *in vitro* have shown ADM and mAMSA to have similar effects, which has led to the suggestion that mAMSA may be an alternative to ADM in the clinic (Tobey *et al.*, 1978).

The present work was undertaken to compare directly the toxicity of ADM and mAMSA to mammalian cells *in vitro*. This was done using V79 cells with differing culture histories and a cell line with induced resistance to ADM. The response of cells to ADM and mAMSA after treatment with misonidazole was also examined, since in previous studies where misonidazole has been used in combination with alkylating agents a synergistic cytotoxicity has been seen (Rose *et al.*, 1980; Stratford *et al.*, 1980). Finally, sister-chromatid exchanges (SCEs) were used to indicate DNA damage caused by each drug.

### MATERIALS AND METHODS

*Cells.*—Chinese hamster V79-379A cells were used in most of these experiments. Cells were routinely grown at 37°C in 250ml conical flasks in Eagle's minimal essential medium (MEM) modified for suspension cultures (Flow Laboratories Ltd) and supplemented with 7·5% foetal calf serum (FCS) (Gibco-Biocult Ltd). Cells were maintained in log-phase growth at concentrations varying between $10^5$ and $10^6$ cells per ml. A doubling time of 10–12 h required cells to be diluted daily. V79-379A cells were also grown in monolayer culture in MEM plus 15% FCS, and passaged twice weekly. All medium containing cells was buffered with bicarbonate to pH 7·4. For most experiments cells were harvested from asynchronous exponentially growing cultures.

Some experiments were done with cells with a history of chronic hypoxia, plateau-phase cells, cells cultured at pH 6·8 or thermotolerant cells. Cells with a history of chronic hypoxia were prepared by taking log-phase cells and holding them for 16 h under anaerobic conditions (Smith *et al.*, 1980; Rajaratnam *et al.*, 1981). Plateau-phase cells were taken from unfed suspension cultures which became density inhibited at a cell concentration of $1 \cdot 2–1 \cdot 6 \times 10^6$ cells/ml. Under these conditions, the pH of the culture medium had been reduced to around 6·9. Other experiments involving reduced pH were done with exponentially growing cells which had been cultured in medium at pH 6·8 for 16 h before drug treatment. Thermotolerant cells were prepared by maintaining log-phase cells at 41°C (pH 7·4) for 16 h (Stratford *et al.*, 1981*b*). V79 cells cultured under conditions of either reduced pH or 41°C are still capable of progression, with doubling times about 16 and 20 h respectively.

The culture conditions described above were not inimical to our V79 cells, and all these cell populations had plating efficiencies $\geqslant 90\%$ before treatment with cytotoxic drugs.

An ADM-resistant Chinese hamster cell line (77A-177, Belli & Harris, 1979; Belli, 1979) was sometimes also studied. These cells had originally been exposed to 0·1$\mu$M ADM for 77 weeks, followed by a further 177 weeks growth in drug-free medium, during which time they retained their resistance to ADM. In our experiments they were maintained in exponential phase in monolayer culture in MEM plus 15% FCS.

*Cytotoxicity studies.*—Cells at $\sim 5 \times 10^5$/ml were transferred to plastic universal containers with the required amount of drug to give a final volume of 10 ml. The containers were placed in a shaking water bath at 37°C in air. After 1 h the cells were removed, washed by centrifugation and resuspension, counted, diluted and plated in MEM plus 15% FCS. They were then incubated for 7 days at 37°C before staining in methylene blue and scoring for colony formation. All colonies visible at $\times 2$ magnification ($\sim 50$ cells or more) were counted.

Some experiments involved pre-treatment with misonidazole (Stratford *et al.*, 1980). Exponentially growing cells were exposed to 5mM misonidazole at 37°C for 2 h under hypoxic conditions, before treatment in air with mAMSA or ADM.

The 77A-177 (ADM-resistant) cells were treated with ADM or mAMSA as follows. Cells were harvested from a monolayer by washing with 0·02% EDTA and 0·08% trypsin, counted, diluted and plated on to 6cm plastic Petri dishes in a total volume of 2 ml MEM plus 15% FCS. The cells were left to attach for $2\frac{1}{2}$ h, the medium aspirated from the dishes and replaced with 2 ml fresh medium containing either mAMSA or ADM. Dishes were incubated for 1 h at 37°C, aspirated, replenished with 2 ml fresh growth medium and incubated for 7 days. As a control, parallel experiments were conducted in an identical fashion using V79-379A cells.

*SCE experiments.*—Asynchronous, exponential V79-379A monolayer cultures were grown in the presence of 10$\mu$M 5-bromodeoxyuridine (BrdU, Sigma) for 18 h in darkness (Ben-Hur & Elkind, 1972). During the first hour the cultures were treated with ADM or mAMSA. Colcemid (CIBA), at a final concentration of $10^{-6}$M, was added for the final 2 h of the BrdU exposure. The cells were harvested by trypsinization, resuspended in 0·075M KCl for 15 min and fixed with methanol:glacial acetic acid (3:1). Air-dried slides were stained in 0·4 mg/ml Hoechst 33258 for 4 h, rinsed in distilled water and mounted in 0·16M disodium orthophosphate and 0·04M trisodium citrate (pH 8·8). The slides were exposed for 45 min to fluorescent light from a Philips CS200W-4 mercury lamp mounted on a Reichert "Polyvar" microscope and filtered with a Reichert "V2" excitation filter. Finally, the slides were rinsed and restained in 4% Giemsa for 5 min.

*Compounds.*—mAMSA, supplied by the late Dr B. F. Cain (Auckland Division, Cancer Society of New Zealand) was made up in distilled water immediately before use. Concentrations were measured spectrophotometrically at 433 nm. Adriamycin (ADM) available in 10mg sterile vials (Montedison Pharmaceuticals Ltd) was reconstituted in 5 ml sterile distilled water and diluted with PBS. Misonidazole was supplied by Dr Carey

FIG. 1.—Effect of a 1h exposure of ADM or mAMSA on the survival of exponentially growing V79-379A cells in air at 37°C. Points represent the average of 5 experiments and error bars the spread of the data.

Smithen of Roche Products Ltd, Welwyn Garden City, Herts, and was made up in MEM plus 7·5% FCS on the day required.

RESULTS

ADM and mAMSA show dose-dependent cytotoxicity to exponentially growing, aerobic V79-379A cells at 37°C (Fig. 1). The survival curve for mAMSA shows that for each increment in drug dose the corresponding decrease in survival is smaller. The shape of the survival curve for ADM is basically the same as for mAMSA, except that at low doses there is an apparent shoulder. Similar results for exponentially growing V79 cells exposed to ADM or mAMSA have been obtained by Belli & Harris (1979) and Wilson *et al.* (1981).

Figs 2 and 3 show the cytotoxicity of ADM and mAMSA to plateau-phase cells, cells rendered chronically hypoxic, thermotolerant cells and cells grown at pH 6·8. When compared to data for exponentially growing cells at pH 7·4 (Fig. 1) it is evident that, for treatment with either drug:

1. Plateau-phase cells and cells rendered chronically hypoxic before drug treatment in air are resistant to drug action;
2. After prolonged exposure of exponentially growing cells to reduced medium pH, the cytotoxicity is reduced, but this is not sufficient to account wholly for the decreased sensitivity of plateau-phase cells; and
3. There is no change in the drug sensitivity of thermotolerant cells.

Fig. 4 shows results comparing the effect of the two drugs on the ADM-resistant 77A-177 cells. It is clear that this cell line is cross-resistant to mAMSA.

We have carried out experiments to ascertain whether misonidazole potentiates the effect of the intercalating agents

FIG. 2.—Effect of 1h exposure to ADM in air at 37°C on the response of plateau-phase cells, cells rendered chronically hypoxic, thermotolerant cells, and cells grown at pH 6·8. Points represent the average of 3 experiments and error bars the spread of the data. Points alone show the mean of two experiments.

ADM and mAMSA in the same way as with alkylating agents (Rose *et al.*, 1980; Stratford *et al.*, 1980). Fig. 5 shows that pretreatment with misonidazole in $N_2$ (which alone reduces survival to around 0·6) protects against the subsequent exposure to ADM or mAMSA. For 10$\mu$M ADM the survival is increased 10-fold,

FIG. 3.—Effect of 1h exposure to mAMSA in air at 37°C on the response of plateau-phase cells, cells rendered chronically hypoxic, thermotolerant cells, and cells grown at pH 6·8. Points represent the average of 3 experiments and error bars indicate the spread of the data.

FIG. 4.—Survival curves for V79-379A (●) and 77A-177 cells (○) exposed to ADM or mAMSA for 1 h in air at 37°C. Points represent the mean of 2 experiments.

whereas for 10μM mAMSA survival is increased 100-fold. The cytotoxicity of ADM or mAMSA was unaffected, whether the pretreatment was with misonidazole in air for 2 h or with $N_2$ alone for 2 h (data not shown).

A comparison of the DNA-damaging effects of ADM and mAMSA was carried out using SCE as an end-point. The frequencies of SCE as a function of subtoxic doses of the drugs are summarized in the Table, and dose–response curves are illustrated in Fig. 6. Fig. 7 shows a chromosome spread for a cell treated with 10μM BrdU for 18 h; SCE were scored by counting the exchange sites (arrows in Fig. 7). Fig 8 and 9 show the distribution of SCE per cell within the dividing population after treatment with either ADM or mAMSA. There is a dose-dependent increase in the incidence of SCE after treatment with either drug, though at higher concentrations ADM appears to be slightly more effective.

SCE experiments involved cells being treated with 10μM BrdU for 18 h. In drug-free controls it was found that this treatment alone reduced the surviving fraction to ~0·8. However, little further reduction in the surviving fraction is seen when a 1h exposure to 0·1 and 0·2μM of either drug is given in combination with BrdU treatment.

### DISCUSSION

In this study we have set out to compare *in vitro* the cytotoxic and DNA-damaging effects of two intercalating agents, ADM and mAMSA. Both drugs produce survival curves of similar shape for exponentially growing cells. It is not clear from our data whether the shape of the survival curves is due to heterogeneity of drug sensitivities.

FIG. 5.—Survival curves of V79-379A cells treated with 5mM misonidazole for 2 h under hypoxic conditions before a 1h drug exposure to ADM or mAMSA, in air at 37°C. Points represent the average of 3 experiments and error bars the spread of the data. Dashed lines are the survival curves in the same experiment for cells un-pretreated.

TABLE.—*Frequency of SCE in ADM- and mAMSA-treated cells. 100 well-spread metaphases with 22 chromosomes were scored for each drug concentration*

| Drug dose ($\mu$M) | | SCE metaphase ±s.e. | Range |
|---|---|---|---|
| Control | | 6·7±0·3 | 1–12 |
| mAMSA | 0·05 | 10·7±0·4 | 2–22 |
| | 0·10 | 15·6±0·7 | 1–33 |
| | 0·15 | 18·0±0·6 | 6–34 |
| | 0·20 | 22·8±0·9 | 6–44 |
| ADM | 0·05 | 10·7±0·4 | 3–20 |
| | 0·10 | 16·6±0·5 | 6–27 |
| | 0·15 | 22·7±0·7 | 10–46 |
| | 0·20 | 27·3±0·9 | 12–56 |

However, it has been suggested by others (Belli & Piro, 1977) that at high concentrations ADM may saturate target binding sites. This model is consistent with the shapes of the dose–response curves for ADM, and a similar mechanism may apply to mAMSA.

There is considerable difference between the drug sensitivities of plateau-phase and log-phase cells. For 10$\mu$M ADM, surviving fractions are $0{\cdot}4 \times 10^{-4}$ and 0·25 for exponentially growing and plateau-phase cells respectively, and for a similar concentration of mAMSA the surviving fraction of these cell populations are $0{\cdot}2 \times 10^{-3}$ and $0{\cdot}2 \times 10^{-1}$. This indicates that the differential in sensitivity between cycling and non-cycling cells may be somewhat greater for ADM. It has been shown previously that there is little difference in the sensitivity of cycling and non-cycling cells towards mAMSA when iso-leucine starvation is used to produce non-cycling cultures (Tobey *et al.*, 1978). Further, Tobey *et al.* (1976) showed that the differential toxicity of ADM for cycling *vs* non-cycling cells (produced by iso-leucine starvation) is much less than that reported here and in the work of others (Barranco *et al.*, 1974; Twentyman, 1976). Non-cycling cells produced by Tobey *et al.* (1976, 1978) compared with plateau-phase cells induced as described

FIG. 6.—The relationship between drug dose and SCE frequency. Points represent the mean of 100 metaphases scored from at least 3 separate experiments and error bars indicate s.e.

in this work may represent different growth-arrested states. Cells harvested from exponentially growing cultures and held for 16 h under hypoxic conditions experience growth arrest, and become resistant to ADM (Smith *et al.*, 1980). These cells are also more resistant than exponentially growing cells to mAMSA. Cells held at 41°C for long enough to modify their response to heat damage (Stratford *et al.*, 1981*a*) or the cytotoxic action of some drugs (Stratford *et al.*, 1981*b*) continue cycling, and this in turn is reflected in these cells having similar sensitivities to ADM and mAMSA when compared to exponentially growing cells held constantly at 37°C. Taken together these results are consistent with the proposal that the growth status of cells *in vitro* will be an important determinant of sensitivity to ADM or mAMSA.

Cells held at pH 6·8 for 16 h before drug treatment continue cycling. However, the cells at this pH are slightly more resistant to both ADM and mAMSA when compared to exponentially growing cells held at pH 7·4. Both mAMSA and ADM are weak bases, with pKa values of 7·3 (Wilson *et al.*, 1981) and 8·2 (Skovsgaard, 1977) respectively. Changes in drug uptake with associated changes in toxicity have been reported as a function of pH for cells exposed to ADM (Skovsgaard, 1977). Further, the cytotoxicity of mAMSA has been reported to be maximal at pH 7·2 (Wilson *et al.*, 1981). The pH range 6·8–7·4 spans that in which tumour cells may be found *in vivo* and it may be important that at these values of pH the cytotoxic actions of ADM and mAMSA are similar.

The basic similarities between the cytotoxic effects of ADM and mAMSA are further demonstrated by the fact that a cell line with induced resistance to ADM shows cross-resistance to mAMSA. This cell line has also been shown to be cross-resistant to Actinomycin D (Belli & Harris, 1979) which has been attributed to genetically controlled membrane changes resulting in decreased drug uptake (Harris *et al.*, 1979).

Cells given 5mM misonidazole for 2 h in $N_2$ before exposure to either ADM or mAMSA become resistant to the cytotoxic actions of both intercalating agents. There is no such effect if cells are pretreated with 5mM misonidazole in *air* for 2 h, nor when cells are given $N_2$ for 2 h with no misonidazole. The exposure to misonidazole in $N_2$ reduces survival to 0·6; it is known that misonidazole shows cell-cycle specificity, in its cytotoxic action with cells in early S being most sensitive and late S/$G_2$ cells most resistant (Whitmore & Gulyas, 1980; Stratford, 1980). The published data on the age responses of cells to ADM and mAMSA have yielded differing results (Kim & Kim, 1972; Barranco, 1975; Bhuyan *et al.*, 1980; Deaven *et al.*, 1978; Roberts & Millar, 1980). However, preliminary work with synchronized V79 cells (West, unpublished)

FIG. 7.—Chromosomes of V79-379A cells labelled with 10 μM BrdU showing "harlequinized" sister chromatids. SCEs are scored by counting the sites of each exchange (arrows).

has indicated that *both* intercalating agents may be most effective in early S. Therefore, our results may indicate that the observed "resistance" to ADM and mAMSA is due to a cell-cycle redistribution caused by misonidazole pretreatment in $N_2$. The somewhat greater resistance to mAMSA than to ADM may be due to subtle differences in age response between these two drugs. An alternative explanation for these results would be that the exposure of cells to misonidazole in $N_2$ alters the cells' membrane properties or metabolic state. Such changes are known when cells are given 5mM misonidazole for 14 h in *air*, and these cells also become more resistant to ADM (J. Belli, personal communication). However, at present we are not able to state categorically which of these mechanisms is operating when cells are given ADM or mAMSA immediately after hypoxic exposure to 5mM misonidazole.

Previous studies have shown that ADM and other intercalating agents can profoundly increase the frequency of SCE in CHO cells (Au *et al.*, 1981; Galloway & Wolff, 1979; Perry & Evans, 1975). Raj & Heddle (1980) have demonstrated that, in general, intercalating agents are efficient inducers of SCE, and the present work confirms these results for V79 cells exposed to ADM. Concentrations of ADM and mAMSA between 0·05 and 0·2 μM have been used to follow the induction of SCE. Although these drug doses produce no significant cytotoxicity as measured by the clonogenic assay, we cannot, on the strength of

FIG. 8.—The effect of varying concentrations of ADM on the induction of SCE in V79–379A cells (1h exposure in air at 37°C).

FIG. 9.—The effect of varying concentrations of mAMSA on the induction of SCE in V79–379A cells (1h exposure in air at 37°C).

these data alone, deduce whether this end-point may be related to SCE induction. However, it is apparent that the magnitude of SCE formation by each drug is similar (*e.g.* at 0·1 μM the SCE per cell is 15·6 ± 0·7 and 16·6 ± 0·5 for mAMSA and ADM respectively, compared to only 6·7 ± 0·3 for untreated controls). Less SCE was found in cells exposed to 0·15μM or 0·2μM mAMSA than in those treated with the equivalent concentration of ADM (Fig. 6). The data illustrated in Figs 8 and 9 show that at these drug levels there is a wide distribution of the numbers of SCE scored, with no well-defined mode, after 0·2μM mAMSA. This change in the form of the distribution may result from slight variation in cycle-stage sensitivity to drug action, and therefore the difference between ADM and mAMSA at this dose level may not be significant. Further, the proportion of non-differentially stained mitoses (1st divisions) appeared higher after treatment with 0·2μM mAMSA than after the corresponding dose of ADM, suggesting that at these doses mAMSA may induce more mitotic delay than ADM. Taking these qualifications into account, it is nevertheless apparent that ADM and mAMSA induce SCE at a similar frequency.

In conclusion, we have demonstrated that the DNA damaging and cytotoxic effects of ADM and mAMSA are broadly similar, under a variety of test conditions *in vitro*. We suggest, therefore, that should any difference in effect be seen for these drugs *in vivo* or indeed clinically, this difference will have its origins in the pharmacology of the two drugs, *i.e.* their uptake, tumour penetration, metabolism and excretion, rather than in inherent differences in cell sensitivity.

We wish to thank Dr Jim Belli for generously providing the ADM-resistant cell line; Drs W. Wilson, G. Whitmore and R. Hill for giving us considerable unpublished information; Professor G. Adams and Dr S. Revell for many helpful discussions, and Christine Williamson for excellent technical assistance. The provision of postgraduate studentships (CW and ES) by the MRC is gratefully acknowledged.

### REFERENCES

ATWELL, G. J., CAIN, B. F. & SEELYE, R. N. (1972) Potential antitumour agents. 12.9-acridinylmethane-sulfonanilides. *J. Med. Chem.*, **15**, 611.

AU, W. W., BUTLER, M. A., MATNEY, T. S. & LOO, T. I. (1981) Comparative structure-genotoxicity study of three aminoanthraquinone drugs and doxorubicin. *Cancer Res.*, **41**, 376.

BARRANCO, S. C. (1975) Review of the survival and cell kinetic effects of Adriamycin (NSC-123127) on mammalian cells. *Cancer Chemother. Rep.*, **6**, 147.

BELLI, J. A. (1979) Radiation response and adriamycin resistance in mammalian cells in culture. *Front. Radiat. Ther. Oncol.*, **13**, 9.

BELLI, J. A. & HARRIS, J. R. (1979) Adriamycin resistance and radiation response. *Int. J. Radiat. Oncol. Biol. Phys.*, **5**, 1231.

BELLI, J. A. & PIRO, A. J. (1977) The interaction between radiation and adriamycin damage in mammalian cells. *Cancer Res.*, **37**, 1624.

BEN-HUR, E. & ELKIND, M. M. (1972) Survival response of asynchronous and synchronous Chinese hamster cells exposed to fluorescent light following 5-bromodeoxyuridine incorporation. *Mutation Res.*, **14**, 237.

BHUYAX, B. K., BLOWERS, C. L. & SHUGARS, K. D. (1980) Lethality of Nogalamycin, Nogalamycin Analogs, and Adriamycin to cells in different cell cycle phases. *Cancer Res.*, **40**, 3437.

CARTER, S. K. (1975) Adriamycin: A review. *J. Natl Cancer Inst.*, **55**, 1265.

DEAVEN, L. L., OKA, M. S. & TOBEY, R. A. (1978) Cell-cycle specific chromosome damage following treatment of cultured Chinese hamster cells with 4′-[(9-acridinyl)-amino] methanesulphon-m-anisidide-HCl. *J. Natl Cancer Inst.*, **60**, 1155.

GALLOWAY, S. M. & WOLFF, S. (1979) The relation between chemically-induced sister-chromatid exchanges and chromatid breakage. *Mutat. Res.*, **61**, 297.

GORMLEY, P. E., SETHI, V. S. & CYSYK, R. L. (1978) Interaction of mAMSA with DNA. *Cancer Res.*, **38**, 1300.

HARRIS, J. R., TIMBERLAKE, N., HENSON, P., SCHIMKE, P. & BELLI, J. A. (1979) Adriamycin uptake in V79 and Adriamycin-resistant Chinese hamster cells. *Int. J. Radiat. Oncol. Biol. Phys.*, **5**, 1235.

KIM, S. H. & KIM, J. H. (1972) Lethal effects of Adriamycin on the division cycle of HeLa cells. *Cancer Res.*, **32**, 323.

LEGHA, S. S., GUTTERMAN, J. V., HALL, S. W. & 4 others (1978) Phase 1 clinical investigation of 4′-(9-acridinylamino)methanesulfon-m-anisidide (NSC 249992), a new acridine derivative. *Cancer Res.*, **38**, 3712.

LEGHA, S. S., BLUMENSCHEIN, G. R., BUZDAR, A. U., HORTOBAGYL, G. N. & BODEY, G. P. (1979) Phase 11 study of 4′-(9-acridinylamino)methanesulfon-m-anisidide (AMSA) in metastatic breast cancer. *Cancer Treat. Rep.*, **63**, 1961.

PERRY, P. & EVANS, H. J. (1975) Cytological detection of mutagen-carcinogen exposure by sister-chromatid exchange. *Nature*, **258**, 121.

RAJ, A. S. & HEDDLE, J. A. (1980) Simultaneous detection of chromosomal aberrations and sister-chromatid exchanges. Experience with DNA intercalating agents. *Mutat. Res.*, **78**, 253.

RAJARATNAM, S., SMITH, E., STRATFORD, I. J. & ADAMS, G. E. (1981) Thermotolerance in Chinese hamster cells heated under oxic conditions following chronic culture under hypoxia. *Br. J. Cancer*, **43**, 551.

ROBERTS, P. B. & MILLAR, B. C. (1980) Enhanced killing of mammalian cells by radiation combined with mAMSA. *Br. J. Cancer*, **42**, 684.

ROSE, C. M., MILLAR, J. L., PEACOCK, J. H., PHELPS, T. A. & STEPHENS, T. C. (1980) Differential enhancement of melphalan cytotoxicity in tumour and normal tissue by misonidazole. *Cancer Management*, **5**, 250.

SCHNEIDER, R., SKLAROFF, R., OCHOA, M. & YOUNG, C. (1979) Phase 1 trial of AMSA (4′(9-acridinylamino)methanesulfon-m-anisidide. *Proc. Am. Assoc. Cancer Res.*, **20**, 114.

SCHNEIDER, R. J., WOODCOCK, T. M. & YAGODA, A. (1980) Phase II trial of 4′(9-acridinylamino)-methanesulfon-m-anisidide (AMSA) in patients with hypernephroma. *Cancer Treat. Rep.*, **64**, 183.

SKOVSGAARD, T. (1977) Transport and binding of daunorubicin, adriamycin and rubidazone in Ehrlich ascites tumour cells. *Biochem. Pharmacol.*, **26**, 215.

SMITH, E., STRATFORD, I. J. & ADAMS, G. E. (1980) Cytotoxicity of adriamycin on aerobic and hypoxic Chinese hamster V79 cells *in vitro*. *Br. J. Cancer*, **41**, 568.

STRATFORD, I. J. (1980) The development of hypoxic cell sensitizers for clinical use. In *Scientific Foundations of Oncology: Supplement* (Eds Symington & Carter). London: Heinemann. p. 116.

STRATFORD, I. J., ADAMS, G. E., HORSMAN, M. R. & 4 others (1980) The interaction of misonidazole with radiation, chemotherapeutic agents or heat. *Cancer Clin. Trials*, **3**, 231.

STRATFORD, I. J., RAJARATNAM, S., TER HAAR, G. R., WILLIAMSON, C. & ADAMS, G. E. (1981*a*) Enhancement of hyperthermic damage in normal and thermotolerant cells *in vitro* by pre-treatment with misonidazole. *J. Natl Cancer Inst.*, **67**, (In press.).

STRATFORD, I. J., WILLIAMSON, C. & HARDY, C. (1981*b*) Cytotoxic properties of a 4-nitroimidazole (NSC 38087): A radiosensitizer of hypoxic cells *in vitro*. *Br. J. Cancer*, **44**, 109.

TOBEY, R. A., CRISSMAN, H. A. & OKA, M. S. (1976) Arrested and cycling CHO ovary cells as a kinetic model. Studies with ADM. *Cancer Treat. Rep.*, **60**, 1829.

TOBEY, R. A., DEAVEN, L. L. & OKA, M. S. (1978) Kinetic response of cultured Chinese hamster cells to treatment with 4′](9-acridinyl)amino]-methanesulfon-m-anisidide-HCl. *J. Natl Cancer Inst.*, **60**, 1147.

TWENTYMAN, P. R. (1976) Comparative chemosensitivity of exponential versus plateau-phase cells in both *in vitro* and *in vivo* made systems. *Cancer Treat. Rep.*, **60**, 1719.

WHITMORE, G. P. & GULYAS, S. (1980) Lethal and sublethal effects of misonidazole under hypoxic conditions. *Cancer Management*, **5**, 99.

WILSON, W. R., WHITMORE, G. F. & HILL, R. P. (1981) Toxicity of 4′-(-acridinylamino)methane-sulphon-m-anisidide (m-AMSA) in exponential and plateau-phase Chinese hamster cell cultures. *Cancer Res.*, **41**, 2809.

ZUNINO, F., GAMBETTA, R., DI MARCO, A. & ZACCARA, A. (1972) Interaction of Daunomycin and its derivatives with DNA. *Biochem. Biophys. Acta*, **277**, 489.